# Teflon/SiO_2_ Bilayer Passivation for Improving the Electrical Reliability of Oxide TFTs Fabricated Using a New Two-Photomask Self-Alignment Process

**DOI:** 10.3390/ma8041704

**Published:** 2015-04-13

**Authors:** Ching-Lin Fan, Ming-Chi Shang, Bo-Jyun Li, Yu-Zuo Lin, Shea-Jue Wang, Win-Der Lee, Bohr-Ran Hung

**Affiliations:** 1 Graduate Institute of Electro-Optical Engineering, National Taiwan University of Science and Technology, 43 Section 4, Keelung Road, Taipei 106, Taiwan; E-Mails: d10019004@mail.ntust.edu.tw (M.-C.S.); m10019004@mail.ntust.edu.tw (B.-J.L.); huangbr@mail.ntust.edu.tw (B.-R.H.); 2 Department of Electronic Engineering, National Taiwan University of Science and Technology, 43 Section 4, Keelung Road, Taipei 106, Taiwan; E-Mail: yzlin.ntust@gmail.com; 3 Institute of Materials Science and Engineering, National Taipei University of Technology, Taipei 106, Taiwan; E-Mail: sjwang@ntut.edu.tw; 4 Department of Electrical Engineering, Lee-Ming Institute of Technology, New Taipei City 243, Taiwan; E-Mail: leewd@mail.lit.edu.tw

**Keywords:** indium gallium zinc oxide (IGZO), thin film transistors (TFTs), passivation layer, Teflon, SiO_2_

## Abstract

This study proposes a two-photomask process for fabricating amorphous indium–gallium–zinc oxide (a-IGZO) thin-film transistors (TFTs) that exhibit a self-aligned structure. The fabricated TFTs, which lack etching-stop (ES) layers, have undamaged a-IGZO active layers that facilitate superior performance. In addition, we demonstrate a bilayer passivation method that uses a polytetrafluoroethylene (Teflon) and SiO_2_ combination layer for improving the electrical reliability of the fabricated TFTs. Teflon was deposited as a buffer layer through thermal evaporation. The Teflon layer exhibited favorable compatibility with the underlying IGZO channel layer and effectively protected the a-IGZO TFTs from plasma damage during SiO_2_ deposition, resulting in a negligible initial performance drop in the a-IGZO TFTs. Compared with passivation-free a-IGZO TFTs, passivated TFTs exhibited superior stability even after 168 h of aging under ambient air at 95% relative humidity.

## 1. Introduction

Numerous recent studies have focused on metal–oxide semiconductors, such as amorphous indium–gallium–zinc oxide (a-IGZO), because of their high mobility and transparency; these semiconductors have been applied as active channel layers in thin-film transistors (TFTs) [1,2,3]. Regarding conventional silicon-based TFTs, amorphous silicon exhibits low carrier mobility (0.5–1 cm^2^/V∙s), whereas polycrystalline silicon requires high-temperature fabrication and has problems associated with its nonuniform grain size [4,5]. By contrast, a-IGZO TFTs can be fabricated on plastic substrates at low temperatures and exhibit excellent and uniform electrical characteristics [6,7].

In general, a-IGZO TFTs applied in active-matrix liquid-crystal displays and active-matrix organic light-emitting diodes are typically fabricated using a back-channel-etching structure and five photomasks. To reduce the fabrication cost, Uhm *et al.* proposed a two-photomask scheme in which a gray-tone photomask was used to fabricate TFT devices [8]; however, the lack of an etching-stop (ES) layer damages the a-IGZO active island when source/drain (S/D) electrodes are etched. In a typical process, an additional photomask step is required for creating an ES pattern, presenting a trade-off between fabrication cost and device stability. Therefore, Geng *et al.* [9] proposed using backside-ultraviolet (BUV) exposure through a metal gate electrode to define the ES area; this process reduces the misalignment margin and fabrication cost. However, during ES deposition, the process gas of hydrogen-based materials affects the a-IGZO active layer, thereby increasing the leakage current [10]. To promote device stability, [11] proposed a two-photomask scheme that combines BUV exposure and liftoff schemes for fabricating self-aligned TFT devices. S/D electrode etching is replaced by a liftoff technique, and a low-damage device can thus be obtained.

The a-IGZO TFTs must be passivated to elongate their lifetimes by protecting the metal–oxide semiconductors from ambient air. SiO_2_ is a widely used passivation material in solid-state electronic and optoelectronic devices because of its excellent oxygen and moisture barrier performance [12]. Moreover, SiO_2_ deposition is compatible with conventional large-area deposition processes, such as plasma-enhanced chemical vapor deposition (PECVD) and radio-frequency (RF) sputtering. However, the deposition techniques are based on a plasma process harmful to the metal–oxide materials [13]. Therefore, an appropriate buffer layer must be embedded between the metal–oxide semiconductor and the SiO_2_ passivation layer to protect the a-IGZO TFTs from plasma damage during the deposition. In our previous study [14], noncharged polytetrafluoroethylene (Teflon) was used as the buffer layer and combined with a SiO_2_ barrier layer to provide bilayer passivation for organic TFTs (OTFTs). Teflon is a nonpolar polymer with numerous excellent properties, such as gas and moisture barrier properties, chemical resistance, thermal resistance, and electric insulation. Teflon can be readily deposited through thermal evaporation at low evaporation temperatures, thus preventing thermal stresses from damaging the underlying organic semiconductor during evaporation [15,16]. No initial performance drop in the OTFTs was evident after Teflon/SiO_2_ passivation, and Teflon effectively protects the OTFTs from plasma damage during SiO_2_ deposition.

In the current study, bilayer passivation using Teflon and SiO_2_ is proposed for improving the reliability of a-IGZO TFTs fabricated through a two-photomask self-alignment process. Teflon was deposited as the buffer layer through thermal evaporation; this layer exhibited favorable compatibility with the underlying IGZO channel layer. Furthermore, the effect of moisture on the reliability of the a-IGZO TFTs was substantially reduced after the Teflon/SiO_2_ passivation. The proposed bilayer Teflon/SiO_2_ passivation can be applied to low-cost a-IGZO TFTs fabricated using a two-photomask self-alignment process for improving the reliability of the TFTs.

## 2. Device Fabrication

**Figure 1:**
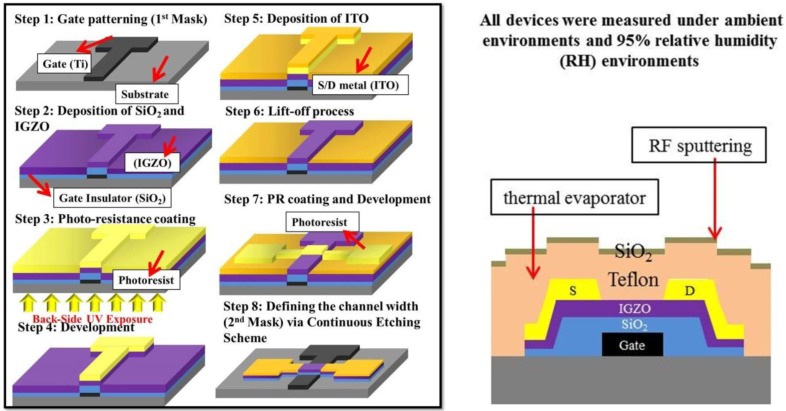
Two-photomask process flow of amorphous indium–gallium–zinc oxide (a-IGZO) thin-film transistors (TFTs) subjected to Teflon/SiO_2_ passivation.

Figure 1 illustrates the proposed two-photomask process for fabricating a-IGZO TFTs. A 160-nm-thick Ti layer was first deposited onto a glass substrate through thermal evaporation, and the layer was patterned to create the gate electrode by using the first photomask. A 200-nm-thick SiO_2_ layer was subsequently deposited using PECVD at 300 °C to create the gate insulator. Next, a 20-nm-thick a-IGZO layer (In_2_O_3_:Ga_2_O_3_:ZnO = 1:1:1 mol%) was deposited through RF sputtering at 200 °C, as follows. First, a photoresist was spin-coated onto an IGZO layer and subjected to BUV exposure by using the Ti gate as a photomask (Figure 1). Second, a 350-nm-thick indium-tin oxide (ITO) layer was deposited through RF sputtering. The backside-liftoff scheme was subsequently used to define the channel length of the self-aligned structure, and the second photomasks were used to define the channel width. Furthermore, reactive-ion etching with CF_4_ gas was employed to continuously etch ITO, IGZO, and SiO_2_ under 80 mTorr, and the etched TFTs were annealed at 200 °C for 30 min in a vacuum chamber. Third, the passivation layer, composed of Teflon and SiO_2_, was deposited over the TFTs. Teflon (400 nm) was deposited using a thermal evaporator (base pressure 2 × 10^−6^ Torr) with the substrate maintained at room temperature (RT). SiO_2_ (100 nm) was subsequently deposited through RF magnetron sputtering by using a SiO_2_ target at 50 W, 5 mTorr, and RT. The electrical parameters of all TFTs were measured in ambient and 95% relative humidity (RH) environments in a glovebox by using a semiconductor parameter analyzer (HP 4145B).

## 3. Results and Discussion

**Figure 2:**
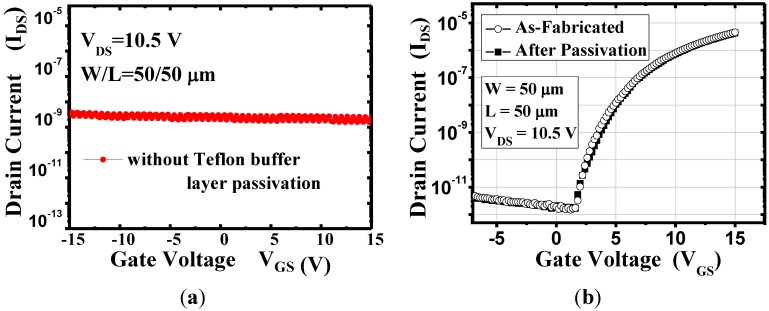
(**a**) Transfer curves (I_DS_–V_GS_) of a-IGZO TFTs without teflon buffer layer; (**b**) Transfer curves (I_DS_–V_GS_) of a-IGZO TFTs before and after Teflon/SiO_2_ bilayer deposition.

Figure 2a shows the transfer characteristic of the device with the SiO_2_ passivation layer only. It is found that the plasma severely damage the device performance after the SiO_2_ deposition. Therefore, the Teflon layer was considered as a buffer layer to suffer the plasma damage from SiO_2_ deposition. Figure 2b illustrates the transfer curves (I_DS_–V_GS_) of the oxide TFTs before and after the Teflon/SiO_2_ bilayer deposition. No change was observed in the transfer curve of the TFTs after the deposition. Crucial electrical characteristics are evident in the transfer curves at an S/D voltage of 10.5 V. The threshold voltages were calculated in the saturation regime by fitting the |I_DS_|^1/2^ *versus* V_GS_ curve of the square law: I_DS_ = μ_FE_C_OX_ (W/2L)(V_GS_ − V_TH_)^2^, where μ_FE_ is the field-effect mobility, C_OX_ is the capacitance density of the gate insulator, V_TH_ is the threshold voltage. The maximum and minimum values of drain current (I_DS_) at a drain voltage (V_DS_) of 10.5 V are designated as I_on_ (on-current) and I_off_ (off-current), respectively [17,18,19]. And the shift of threshold voltage ΔV_TH_ is defined ΔV_TH_ = [(V_TH_ after stress − V_TH_ before stress)/V_TH_ before stress]. Before passivation, the field-effect mobility (μ_FE_) was 8.28 cm^2^/V∙s in the saturation region, the threshold voltage (V_TH_) was 4.81 V, and the on/off current ratio (I_ON_/I_OFF_) was approximately 10^6^. After passivation, the values of μ_FE_, V_TH_, and I_ON_/I_OFF_ were 8.67 cm^2^/V∙s, 5.08 V, and approximately 10^6^, respectively as shown in Table 1. These results show that the thermally evaporated Teflon did not physically or chemically damage the underlying IGZO channel layer because of its low evaporation temperature and chemical inertness, and the Teflon layer can effectively protect the a-IGZO TFTs from plasma damage during the SiO_2_ deposition. Therefore, Teflon can be used as the buffer layer for suppressing the *in situ* degradation of a-IGZO TFTs during SiO_2_ passivation.

**Table 1:** The electrical characteristics of devices before and after Teflon/SiO_2_ bilayer deposition.

Electrical parameters	V_TH_	μ_FE_	S.S.	I_on_/I_off_
Before-passivation	4.81	8.28	0.39	2.9 × 10^6^
After-passivation	5.08	8.67	0.40	2.7 × 10^6^

**Figure 3:**
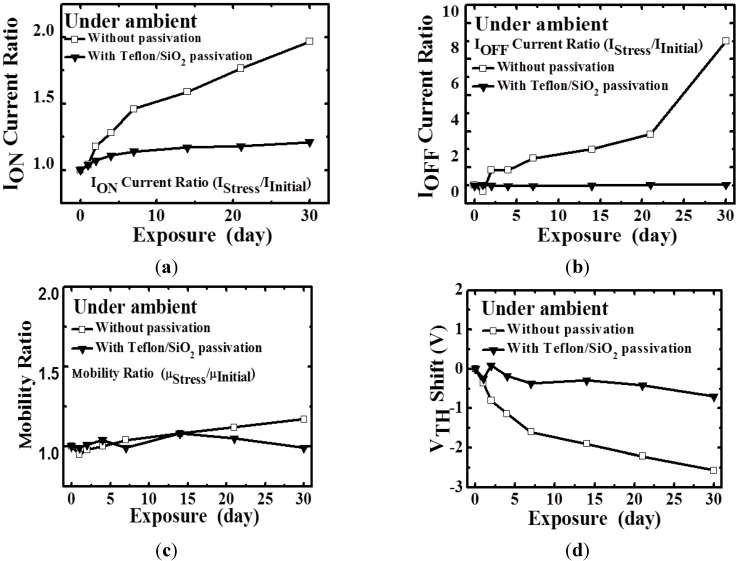
Time-dependent changes in the (**a**) I_ON_; (**b**) I_OFF_; (**c**) μ_FE_; and (**d**) V_TH_ of a-IGZO TFTs with and without Teflon/SiO_2_ passivation in an ambient environment, respectively.

Figure 3 depicts the electrical parameters of the a-IGZO TFTs that were and were not subjected to Teflon/SiO_2_ bilayer passivation in an ambient environment for 30 days. The electrical reliability of the TFTs subjected to Teflon/SiO_2_ passivation was superior to that of the TFTs not subjected to passivation. The markedly negative V_TH_ shift and the increased leakage current (I_off_) in the TFTs not subjected to passivation are attributed to free electrons generated in the IGZO layer during the prolonged stress durations. This result suggests that, as reported previously [20], adsorbed H_2_O donates a partial negative charge to the a-IGZO surface in either molecular or hydroxyl forms. Similarly, the formation of extra electron carriers has been attributed to the donation of electrons (*i.e.*, the donor effect) [20] from chemically adsorbed H_2_O molecules to the surfaces of IGZO. The V_TH_ of TFTs can be expressed as V_TH_ ={[(q × D_bt_ × (E_F_ – E_i_))/C_i_] – (Q_f_/C_i_) + [(q × D_it_ × (E_F_ – E_i_))/C_i_] – (Q_m_/C_i_) – [(q × D_D_ – D_A_)/C_i_] + Δ_MS_} where C_i_ is the insulator capacitance; Q_f_ is the oxide charge density; D_bt_ and D_it_ are the bulk trap density and the interface trap density, respectively, and Δ_MS_ is the work function difference between metal/semiconductor. D_D_ and D_A_ are the donor and acceptor concentrations, respectively [21]. It is described that the carrier concentration in the channel layer influences V_TH_. The extra electrons induced by the H_2_O molecules may form a back channel layer with a high electron concentration, thus producing a more negative voltage that depletes the channel layer.

**Figure 4:**
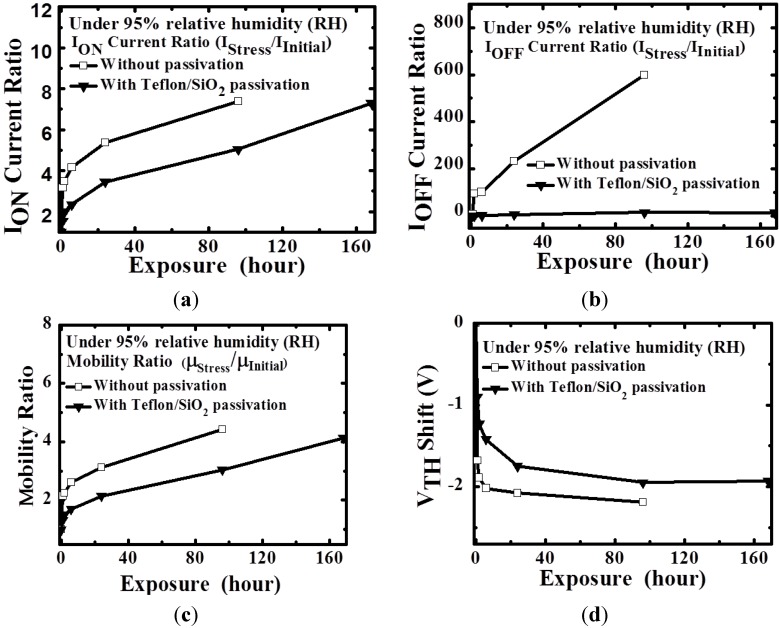
Time-dependent changes in the (**a**) I_ON_; (**b**) I_OFF_; (**c**) μ_FE_; and (**d**) V_TH_ of a-IGZO TFTs with and without Teflon/SiO_2_ passivation in a 95% relative humidity environment, respectively.

**Figure 5:**
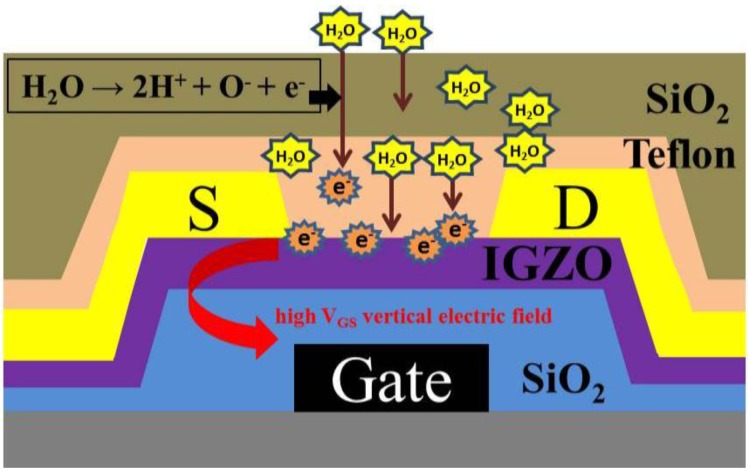
Schematic of H_2_O-molecule-induced extra electron carrier model for a-IGZO TFTs.

Further investigation was required to clarify the effect of moisture on the TFTs. Therefore, the TFTs were placed in a 95% RH environment to induce the effect of moisture. Figure 4 depicts the electrical parameters of a-IGZO TFTs that were and were not subjected to Teflon/SiO_2_ bilayer passivation at 95% RH for 168 h. Both a-IGZO TFTs exhibited increased mobility and I_on_ current ratios. The TFTs not subjected to Teflon/SiO_2_ passivation exhibited considerably more degradation than did those subjected to it. In addition, the device without bilayer passivation had been not the transfer characteristics after the stress in the period of 168 h. Previous studies [20,22] have reported that the extra electron carriers cause a high electron concentration in the back channel, reducing V_TH_; this process can be described as H_2_O → 2H^+^ + O^−^ + e^−^. Therefore, when the TFTs subjected to bilayer passivation were placed in a 95% RH environment, a small amount of H_2_O molecules diffused through SiO_2_ (*i.e.*, the passivation layer) and piled onto the Teflon layer because of the high concentration of H_2_O. The piled H_2_O molecules induce extra electron carriers because of the high vertical electric field (V_GS_), thus causing the negative V_TH_ shift of the TFTs subjected to bilayer passivation. Figure 5 depicts a schematic of an H_2_O-molecule-induced extra electron carrier model for a-IGZO TFTs.

**Figure 6:**
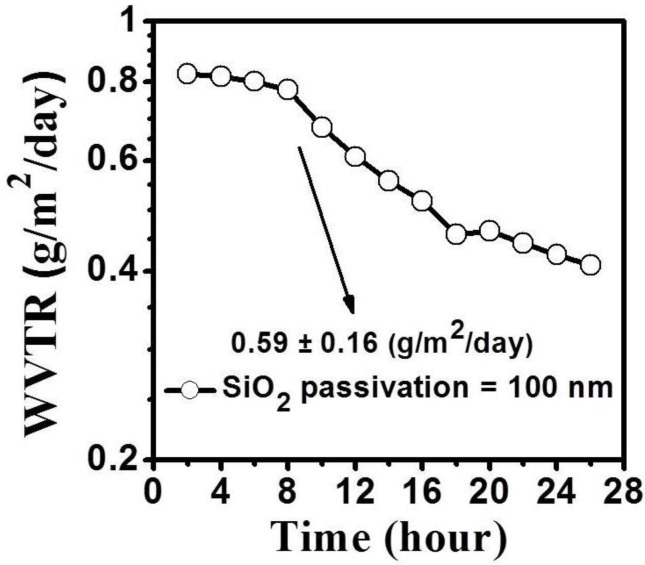
Water vapor transmission rate of polycarbonate substrates with a 100-nm-thick SiO_2_ layer.

To confirm the moisture-barrier property of the RF sputtered SiO_2_ film, the water vapor transmission rate (WVTR) of the 100-nm-thick SiO_2_ layer deposited on one side of a polycarbonate (PC) substrate was investigated. The WVTR measurement was conducted at 38 °C and 100% RH by using a water vapor permeation measurement system (MOCON Aquatran Model 1). As shown in Figure 6, the WVTR mean value of the SiO_2_-coated PC substrate was approximately 0.59 ± 0.16 g/m^2^/day. And for commercially available polymers, such as Polylmide (PI), PolyTetraFlouro Ethylene (Teflon), PolyEthylene Terephthalate (PET) and PolyEthylene Naphthalate (PEN), the permeation rates are typically >1 × 103 g/m^2^/day for oxygen and >1 g/m^2^ day for water at last [23]. In this study, the moisture-blocking layer was SiO_2_ because of its excellent oxygen and moisture barrier performance. The Teflon layer was considered to be a buffer layer suffer the plasma damage from the SiO_2_ layer depositing. In summary, the Teflon/SiO_2_ bilayer passivation effectively blocked moisture diffusion, reducing degradation relative to that of the a-IGZO not subjected to passivation.

## 4. Conclusions

This study demonstrated a bilayer passivation method that involves using a Teflon and SiO_2_ combination layer for considerably improving the reliability of a-IGZO TFTs—Which exhibit a self-aligned structure—Fabricated using a novel two-photomask process. The results show that the electrical performance of the fabricated a-IGZO TFTs subjected to Teflon/SiO_2_ passivation was comparable to that of pristine a-IGZO TFTs. Depositing a Teflon buffer layer did not damage the underlying channel layer; this buffer layer effectively protected the a-IGZO TFTs from plasma damage during SiO_2_ passivation. This study concludes that the proposed bilayer Teflon/SiO_2_ passivation effectively blocks the moisture diffusion, thus yielding superior reliability even after 168 h of aging under ambient air at 95% RH.
